# Kctd9 Deficiency Impairs Natural Killer Cell Development and Effector Function

**DOI:** 10.3389/fimmu.2019.00744

**Published:** 2019-04-10

**Authors:** Xiaoping Zhang, Peng Wang, Tao Chen, Weiming Yan, Xiaoxu Guan, Guanxin Shen, Xiaoping Luo, Xiaoyang Wan, Qin Ning

**Affiliations:** ^1^Institute of Infectious Disease, Tongji Hospital of Tongji Medical College, Huazhong University of Science and Technology, Wuhan, China; ^2^Department of Infectious Disease, Tongji Hospital of Tongji Medical College, Huazhong University of Science and Technology, Wuhan, China; ^3^Department of Immunology, School of Basic Medicine, Tongji Medical College, Huazhong University of Science and Technology, Wuhan, China; ^4^Department of Pediatrics, Tongji Hospital of Tongji Medical College, Huazhong University of Science and Technology, Wuhan, China

**Keywords:** NK cells, Kctd9, fulminant hepatitis, liver damage, development

## Abstract

We previously showed that potassium channel tetramerization domain containing 9 (KCTD9) is aberrantly expressed in natural killer (NK) cells in patients with hepatitis B virus-associated acute-on-chronic liver failure and mice with experimental fulminant hepatitis. However, the mechanism underlying the regulation of NK cell function and fulminant hepatitis progression by KCTD9 is unknown. Here, we investigated the role of Kctd9 in regulation of early development, maturation, and function of NK cells using *Kctd9-*knockout mice. Compared to wild-type mice, *Kctd9*-deficient mice exhibited impaired NK cell lineage commitment, as evidenced by selective reduction in the refined NK progenitors, and incomplete NK cell maturation, as manifested by a higher proportion of CD11b^−^ NK cells and a lower percentage of CD11b^+^ NK cells with high proliferative potential. Moreover, *Kctd9*-depleted NK cells displayed insufficient IFN-γ production, degranulation, and granzyme B production in response to cytokine stimulation, and attenuated cytotoxicity to tumor cells *in vitro*. The defect in NK cells was further supported by ameliorated liver damage and improved survival in *Kctd9*-deficient mice following murine hepatitis virus strain-3 (MHV-3) infection, which otherwise leads to immune-mediated fulminant hepatitis, a phenotype homologous to that caused by NK cell depletion in wild-type mice. Further investigation to identify the underlying mechanism revealed that Kctd9 deficiency hindered the expression of transcription factors, including Ets1, Nfil3, Eomes, and Id2 in NK cells. Collectively, our data reveal that Kctd9 acts as a novel regulator for NK cell commitment, maturation, and effector function.

## Introduction

Natural killer (NK) cells are the major effector lymphocytes of the innate immune system in host defense against tumors and invading pathogens. Complete activation of NK cells which doesn't require prior sensitization is vital for initial combat against infection as well as for activating the adaptive immune response by physical interaction with dendritic cells or secretion of immunoregulatory cytokines.

In adults, NK cell development from common lymphoid progenitors (CLPs) occurs in the bone marrow (BM) through three stages: NK progenitors (NKPs), immature NK (iNK) cells, and mature NK (mNK) cells (1). NKPs were initially defined as CD122^+^NK1.1^−^; however, fewer than 10% of cells with this phenotype give rise to NK cells (2). Therefore, the NKP phenotype has been redefined into refined NKPs (rNKP; CD27^+^CD244^+^CD127^+^CD122^+^CD135^−^) and pre-NKP (CD27^+^CD244^+^CD127^+^CD122^−^CD135^−^) with full potential of NK cell lineage commitment (3, 4). CD3^−^NK1.1^+^ cells acquire expression of several integrins in the order of Alpha V, DX5 and Mac-1 (CD11b) during the maturation process (5). The expression of NK cell receptors such as NKG2, followed by the Ly49 receptors, is initiated in an IL-15-dependent manner after the acquisition of NK1.1 (5, 6). The acquisition of NK1.1 marks the iNK stage, during which the cells undergo a sequential maturation program that includes four discrete steps discriminated by surface expression of CD27 and CD11b (CD11b^−^CD27^−^, CD11b^−^CD27^+^, CD11b^+^CD27^+^, and CD11b^+^CD27^−^) (7). CD11b^−^ NK cells are less mature NK cells and are mainly found in the bone marrow and lymph nodes. S1P5R expression after the acquisition of CD11b enables CD11b^+^ NK cell migration from the bone marrow to the periphery, where they are equally distributed to other organs (8). CD11b^−^CD27^+^NK cells express high levels of NKG2A and low levels of Ly49 receptors; however, CD11b^+^CD27^+^ and CD11b^+^CD27^−^NK cells, which are mainly located in the periphery, are equipped with a full repertoire of Ly49 receptors and cytotoxic potential (9). Another discrepancy between iNK cells and mNK cells is the proliferative capacity, which occurs at a high potential in the immature stages and wanes upon further maturation. Specifically, the proliferating potential during NK cell development continued after acquiring NK1.1 expression and before terminal maturation marked by high CD43 and KLRG1 expression (5, 10).

NK cell development and function depend on IL-15 signaling and spatiotemporal expression of intrinsic transcription factors. IL-15- or IL-15Rα-deficient mice selectively lose CD122^high^ lineages, including NK cells, NKT cells, and memory CD8^+^ T cells (11, 12). Transcriptional factors like ETS1and E4BP4 regulate NK cell lineage commitment, whereas other factors such as Eomes, T-bet, ID2, TOX, IRF2, and GATA3 modulate NK cell maturation (13). Mice lacking ETS1 or NFIL3/E4BP4 have fewer NK cell-committed progenitors in the bone marrow and mNK cells in the periphery, and E4BP4 merely specifies NK cell lineage without affecting NKT cells or T cells (13–15). ETS1 regulates the transcription of genes coding for T-bet and ID2, whereas E4BP4 directs Eomes and ID2 expression during NK cell lineage commitment (14, 15).

Eomes and T-bet are master regulators reciprocally involved in NK cell maturation. Hematopoietic-specific depletion of Eomes results in the selective reduction of DX5^+^ or CD11b^+^ NK cells, with TRAIL^+^DX5^−^immature cells dominating the NK cell population, whereas, T-bet-deficient mice (*Tbx21*^−/−^) contain DX5^+^ NK cells without maintaining Eomes^−^Trail^+^ NK cells (16). Deletion of both results in the absence of NK antigen-expressing cells without affecting CD122^+^ NK cells precursors, suggesting the complementary requirement of Eomes and T-bet for the late development of NK cells. ID2 is required for late maturation, IL-15-dependent metabolism, and survival of NK cells because it regulates IL-15 signaling without affecting the transcription factors required for NK cell development (5, 17). TOX-deficient mice maintained normal numbers of iNK cells but reduced numbers of mNK cells with low ID2 expression (18). Notably, thymic and liver-resident NK cells are of independent origins from bone marrow-derived NK cells (also termed conventional NK cells), and share a developmental program including GATA3 expression distinct from that of conventional NK cells as well (19, 20).

Potassium channel tetramerization domain containing 9 (KCTD9), a pentapeptide repeat-containing protein that mimics DNA structure (21), was first identified to be highly expressed in NK cells in an animal model of viral fulminant hepatitis and in patients with hepatitis B virus-associated acute-on-chronic liver failure (22, 23). Knockdown of KCTD9 in immortalized NK cells impaired cytokine production and cytotoxicity, suggesting its potential role in regulation of NK cells function (23). However, direct evidence of the involvement and mechanism of KCTD9 in NK cell function *in vivo* is not yet available. In this study, we investigated the role of Kctd9 in NK cell commitment, maturation, effector function, and involvement in viral fulminant hepatitis.

## Materials and Methods

### Mice

*Kctd9*^−/−^ mice were created on a BALB/c background by the Beijing Genomic Institute (Shenzhen, China) using transcription activator-like effector nuclease technology. Wild-type BALB/c mice were purchased from Charles River Laboratories (Beijing, China) and used for preliminary *Kctd9*^−/−^ found breeding and control experiments. Wild-type mice were co-housed for 1–2 weeks with *Kctd9*^−/−^ mice to accommodate the environment before use for the experiments. Mice with two genotypes at the same age (6–10 weeks old) were used and 4–8 mice were commonly used for each experiment. Mutant genotype was identified by PCR and immunoblotting. The primers are provided in Supplementary Material. All mice were housed in specific pathogen-free conditions. The animal experiments were approved by the Committees on Animal Experimentation of Tongji Hospital and performed in compliance with the Animal Care and Use Guidelines of Huazhong University of Science and Technology.

### Murine Hepatitis Virus Strain 3 (MHV-3)-Induced Fulminant Hepatitis in BALB/c Mice

MHV-3 was obtained from the American Type Culture Collection (Manassas, VA, USA) and was plaque-purified on a monolayer of delayed brain tumor cells and titer-tested on L2 cells according to a standard plaque assay. Mice were injected intraperitoneally with MHV-3 (100 PFU/mouse) in saline solution (200 μl). For alanine transaminase and aspartate transaminase measurements, blood was collected to isolate serum at the indicated time after virus infection. Ten to twenty mice were used for the infection experiments.

### Cell Preparation

Liver tissue, mesenteric lymph node, and spleen were cut into pieces, and grinded through moisturized 100-μm cell strainer (BD Biosciences, San Jose, CA, USA). Bone marrow were obtained by flushing completed Dulbecco's Modified Eagle Medium (DMEM) through the femurs with a 1-ml syringe. Bone marrow cells were collected by mashing through 100 μm cell strainers. Liver lymphocytes were isolated by following standard protocol (Supplementary Material). For *in vitro* culture treatment, spleen cells were resuspended in lymphocytes separation medium (cat# DKW33-R0100, Dakewe), upon which RPMI 1640 medium were layered. Centrifuged at 800 × g for 20 min and then collected lymphocytes from the interphase. The cells were subjected to red blood cell lysis, except for lymph node cells.

### Flow Cytometry

Cells were stained with Fixable Viability Stain 780 (cat# 565388, BD Biosciences) to facilitate the exclusion of dead cells during analysis. Cells were pre-incubated with Mouse BD Fc Block (clone 2.4G2, cat# 553142, BD Biosciences) before staining. Cells were incubated with antibodies against surface molecules, and then were subjected to permeabilization and intracellular antibody staining. Cells were finally subjected to flow cytometry with a BD FACS Canto II or BD LSR II (BD Biosciences). The procedure is detailed in the Supplementary Material.

### NK Cell Isolation

Untouched NK cells were isolated from splenocytes using magnetic beads for negative selection, according to the manufacturer's instructions of NK Cell Isolation Kit II (cat# 130-096-892, MiltenyiBiotec). Cells achieving>70% purity were applied to functional assay.

### *In vitro* Cell Activation

Splenic lymphocytes (1 × 10^6^) were seeded in RPMI 1640 medium (1 ml) in 12-well plates and treated with recombinant murine IL-12 (1 ng/ml or 5 ng/ml; cat# 210-12, PeproTech,) and IL-18 (10 ng/ml; cat# B002-5, MBL) for 6 h to assess IFN-γ production. To examine degranulation, splenic lymphocytes were treated with IL-12 (10 ng/ml) and IL-18 (10 ng/ml) for 6 h in the presence of PerCP-Cy5.5-conjugated anti-CD107a antibody (10 μl; clone 1D4B, cat# 121625, BioLegend) or an isotype control antibody as previously described (15, 24). To induce Granzyme B production, purified splenic NK cells (2 × 10^5^) were cultured in RPMI 1640 medium (200 μl) in 96-well U-bottom plates in the presence of recombinant murine IL-15 (20 ng/ml; cat# 210-15, PeproTech) for 24 h (15). Protein transport inhibitors GolgiStop (cat# 554724, BD Biosciences) and GolgiPlug (cat# 555029, BD Biosciences) were added 4 h in advance of cell harvest.

### Proliferation

To examine proliferation, purified splenic NK cells were labeled with 5 μM carboxyfluorescein diacetate succinimidyl ester (CFSE; 5 μm; cat# C34554, ThermoFisher Scientific), and then were seeded in 96-well U-bottom plates (2 × 10^5^ cells/200 μl) and cultured in the presence of IL-15 (50 ng/ml) for 3 days.

### Cytotoxicity Assay

Purified splenic NK cells (1 × 10^5^) were mixed with CFSE-labeled Yac-1 cells in U-bottom 96-well plates at various ratios (effector: target ratio, 20:1, 10:1, 5:1, 2:1) and incubated for 4 h. The cell mixtures were harvested for Annexin V staining with the PE Annexin V Apoptosis Detection Kit I (cat# 559763, BD Biosciences).

### Real-Time PCR

Total RNA from purified splenic NK cells was extracted using RNeasy Plus Micro Kit (cat# 74034, Qiagen), and reverse-transcribed using ReverTra Ace qPCR RT Master Mix (cat# FSQ-201, Toyobo, Osaka, Japan). Quantitative PCR was performed with SYBR Green Real-Time PCR Master Mix (cat# QPK-201, Toyobo, Osaka, Japan). The primers used were listed in the Supplementary Material.

### Statistical Analysis

Unpaired Student's *t*-tests (two-tailed) were performed using GraphPad Prism7 software. *p* ≤ 0.05 was considered to be statistically significant for all tests. The stars in the figures correspond to *p*-values as follows: ^*^p ≤ 0.05, ^**^p ≤ 0.005, ^***^p ≤ 0.001, and ^****^p ≤ 0.0001.

## Results

### Kctd9 Deficiency Ameliorated Liver Damage Following MHV-3 Infection

**Figure 1:**
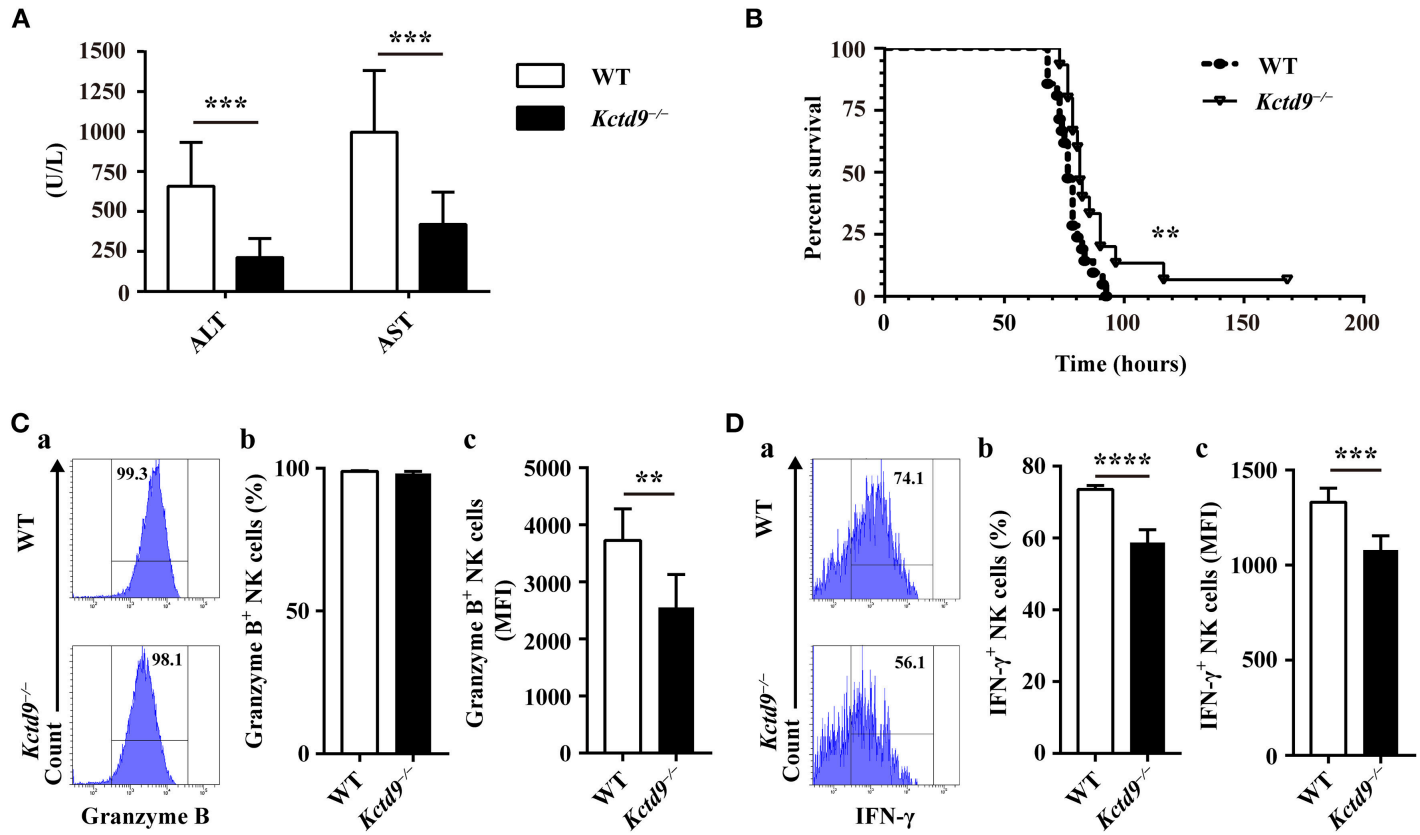
Kctd9 deficiency ameliorated liver damage following MHV-3 infection. Wild-type (WT) and *Kctd9*^−/−^ mice were injected intraperitoneally with MHV-3. **(A)** Serum alanine transaminase (ALT) and aspartate transaminase (AST) levels in WT and *Kctd9*^−/−^ mice 24 h after MHV-3 infection. **(B)** Survival curve of WT and *Kctd9*^−/−^ mice after MHV-3 infection. **(C,D)** Expression of Granzyme B **(C)** and IFN-γ **(D)** by CD3^−^NKp46^+^DX5^+^ NK cells in liver from WT and *Kctd9*^−/−^ mice 48 h after MHV-3 infection. **(a)** Flow cytometric analysis for Granzyme B **(C)** and IFN-γ **(D)**. Plots represent CD3^−^NKp46^+^DX5^+^ NK cells, and frequency of gated population is indicated. **(b,c)** Summary of percentage and intensity of expression of CD3^−^NKp46^+^DX5^+^ NK cells positive for Granzyme B **(C)** and IFN-γ **(D)**. All results were representative of three independent experiments. Ten mice of each genotype were used for measurement of ALT/AST levels, 15 *Kctd9*^−/−^mice and 20-21 WT mice were used for survival experiment, and 4–6 mice of each genotype were used for detection of Granzyme B and IFN-γ in each experiment. Comparison of survival curves: Log-rank (Mantel-Cox) test *P* = 0.0069, Gehan-Breslow-Wilcoxon test *P* = 0.0084; the median survival time: KO: WT 82 h vs. 76.5 h; the survival rate: KO: WT (1/15) vs. 0. Error bars indicate standard deviation. **P < 0.01, ***P < 0.001, and ****P < 0.0001.

We previously revealed the vital contribution of NK cells to liver damage, and the involvement of KCTD9 in NK cell function in viral fulminant hepatitis (22, 25). To verify the requirement of Kctd9 for NK cell effector function *in vivo, Kctd9*^−/−^ mice were used. Forty nucleotides was deleted in the open reading frame of *Kctd9* gene of knockout mice (Supplementary Figure 1A), which may induce frame shift or unspecific splicing of Kctd9 transcript and result in a loss of Kctd9 protein. Mice were infected with MHV-3, which otherwise induces liver damage and fulminant hepatic failure (25, 26). Interestingly, liver damage of *Kctd9*^−/−^ mice was significantly alleviated, as evidenced by lower transaminase levels (Figure 1A). The survival time and survival rate of *Kctd9*-deficient mice were significantly improved, compared to that of the wild-type mice following virus infection (Figure 1B), with less activated NK cells in *Kctd9*^−/−^ mice evidenced by reduced Granzyme B and IFN-γ expression (Figures 1C,D). Collectively, our data suggest that impaired NK cell function contributes to attenuated liver damage in *Kctd9*^−/−^ mice infected with MHV-3.

### Kctd9 Selectively Specifies rNKPs During NK Cell Commitment

As BALB/c mice lack NK1.1, other surface antigens such as NKp46 or DX5 may be used instead of NK1.1 to label committed NK cells. NK precursors generate NK antigen-bearing (NK1.1^+^NKp46^+^) NK cells that can further develop into the mature phenotype and express DX5 and CD11b (5, 7, 27); therefore, we used both NKp46 and DX5 as NK cell markers in our experiments.

**Figure 2:**
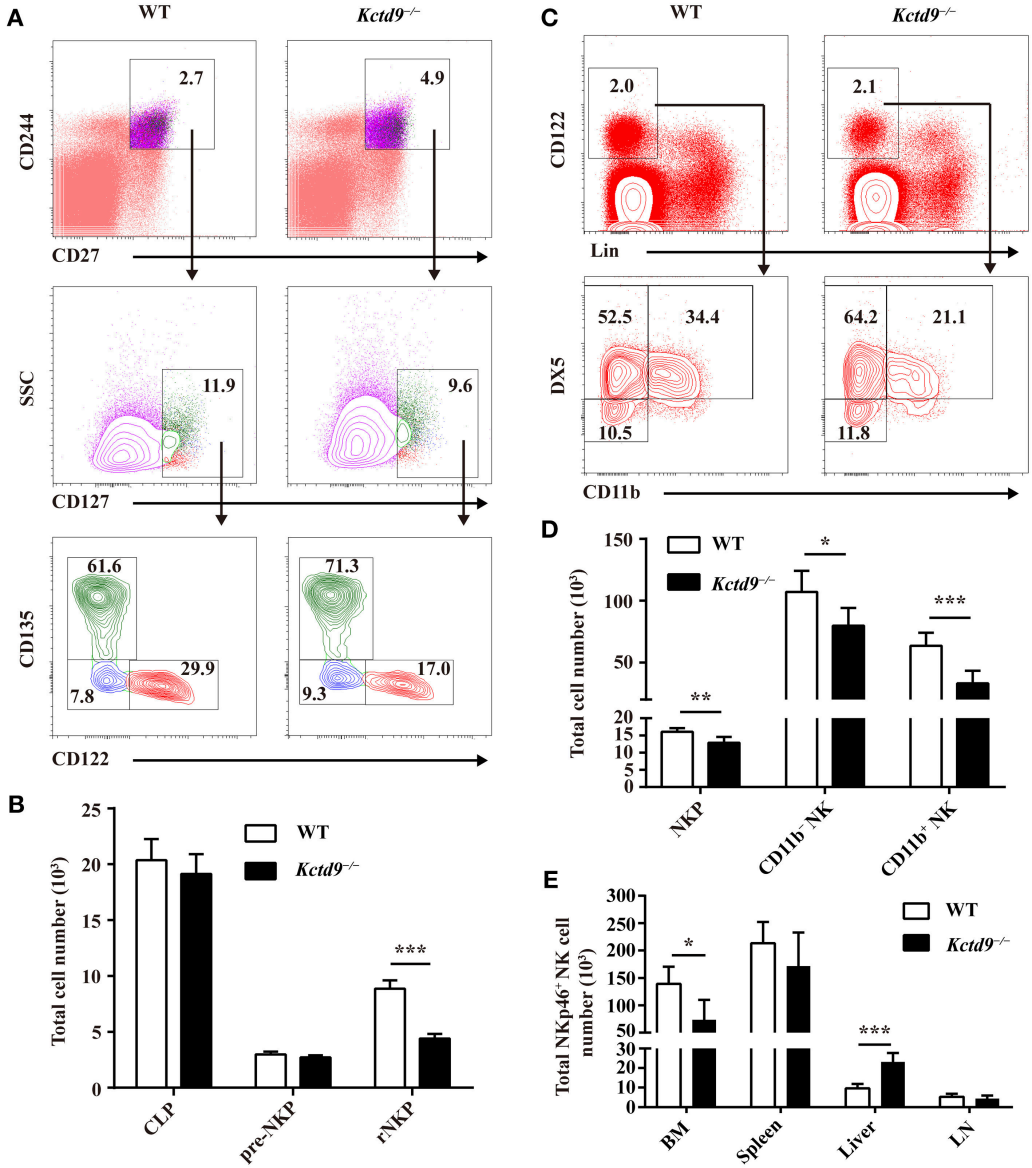
Kctd9 selectively specifies rNKPs during NK cell commitment. **(A**,**C)** Flow cytometry gating strategies for identification of NK progenitors in BM from WT and *Kctd9*^−/−^ mice. **(A)** Using CD135 and CD122 to identify CLP (CD135^+^CD122^−^), pre-NKP (CD135^−^CD122^−^), and rNKP (CD135^−^CD122^+^) among lineage (CD3/CD19/CD11b/Ly6d/DX5)^−^CD27^+^CD244^+^CD127^+^ cells. **(C)** Using DX5 and CD11b to identify NKP (DX5^−^CD11b^−^), CD11b^−^ NK (DX5^+^CD11b^−^), and CD11b^+^ NK (DX5^+^CD11b^+^) cells among lineage (CD3/CD19/CD4/CD8/Ter119)^−^CD122^+^ cells. Frequency of gated population is indicated. **(B**,**D)** The total number of CLP, pre-NKP and rNKP **(B)** or NKP, CD11b^−^ NK, and CD11b^+^ NK cells **(D)** in BM. **(E)** The total number of CD3^−^CD122^+^NKp46^+^ NK cells in the BM, spleen, liver, and mesenteric lymph node (LN) of WT and *Kctd9*^−/−^ mice. All results were representative of three independent experiments. Six–eight mice of each genotype were used in each experiment. Error bars indicate standard deviation. *P < 0.05, **P < 0.01, and ***P < 0.001.

The number of bone marrow cells were not altered between wild-type and *Kctd9*^−/−^ mice (Supplementary Figure 1B). Compared with wild-type mice, *Kctd9*^−/−^ mice exhibited reduced number of rNKPs but not pre-NKPs in bone marrow (Figures 2A,B and Supplementary Figure 1C). Moreover, the number of CLPs, which give rise to a wide spectrum of lymphocytes, including NK cells, was not altered in the bone marrow (Figure 2B). Consistent with the reduction in rNKPs, the numbers of NKPs, CD11b^−^ NK, and CD11b^+^ NK cells in the bone marrow from *Kctd9*^−/−^ mice were also reduced (Figures 2C,D). However, the number of total NK cells in the liver from *Kctd9*^−/−^ mice were higher than that in the wild-type counterparts (Figure 2E). Together, these data suggest that Kctd9 regulates NK cell commitment and maturation.

### Kctd9 Is Required for NK Cells Maturation

**Figure 3:**
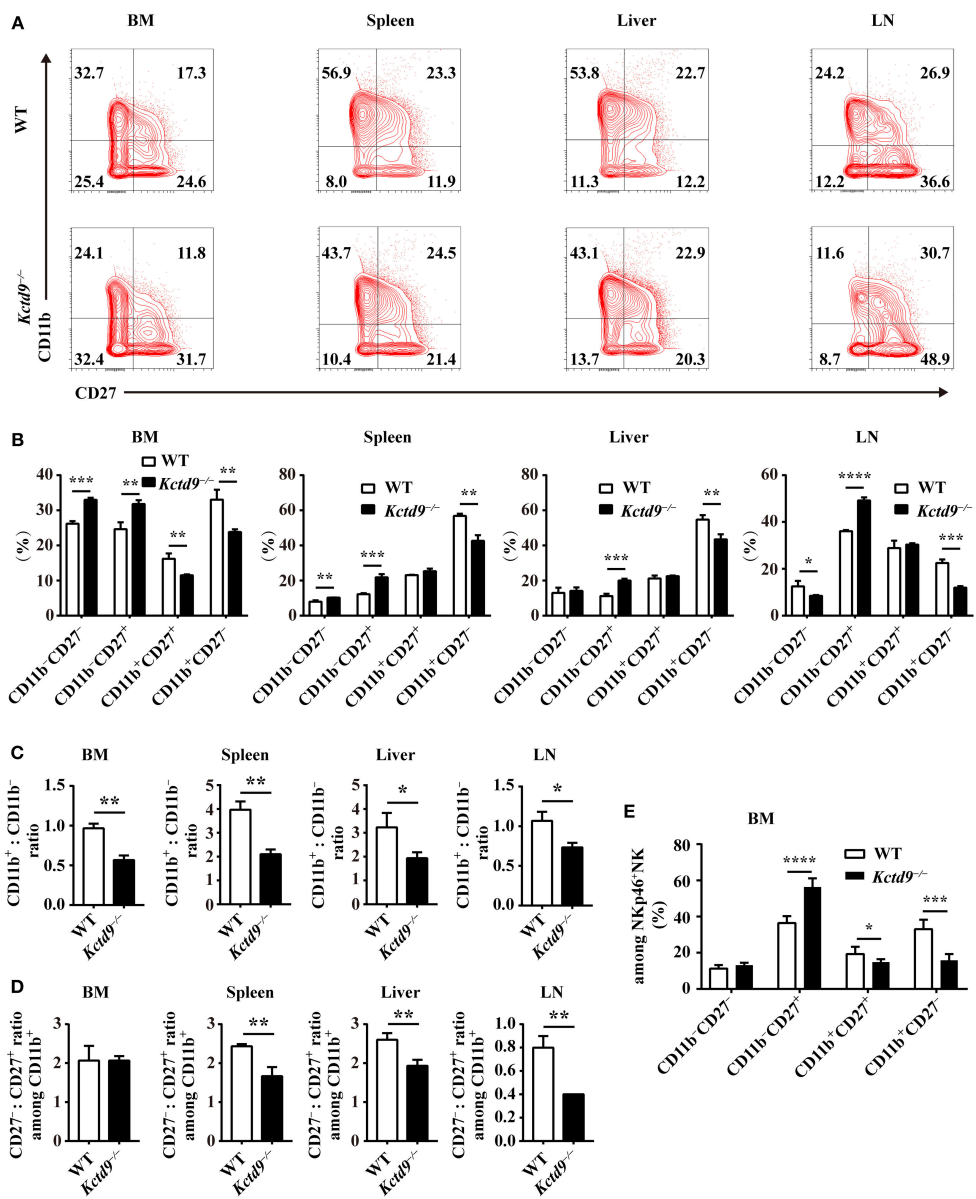
Kctd9 is required for NK cells maturation. **(A)** Flow cytometric analysis for expression of CD11b and CD27 by CD3^−^DX5^+^ cells in four organs from WT and *Kctd9*^−/−^ mice. Numbers in the quadrants indicate the percentage of subsets defined by CD11b/CD27 expression in CD3^−^DX5^+^ cells. **(B)** Summary of the percentages of subsets in CD3^−^DX5^+^ cells. **(C)** The ratio of the CD11b^+^ subset to the CD11b^−^ subset among CD3^−^DX5^+^ cells in indicated organs. **(D)** The ratio of the CD27^−^ subset to the CD27^+^ subset among CD3^−^DX5^+^CD11b^+^ cells in indicated organs. **(E)** The percentages of subsets defined by CD11b/CD27 expression in CD3^−^NKp46^+^ NK cells in BM from WT and *Kctd9*^−/−^ mice. All results were representative of three independent experiments. Four to six mice of each genotype were used in each experiment. Error bars indicate standard deviation. *P < 0.05, **P < 0.01, ***P < 0.001, and ****P < 0.0001.

Development of immature NK cells into mature NK cells falls into a four-stage program identified by surface markers: CD11b^−^CD27^−^, CD11b^−^CD27^+^, CD11b^+^CD27^+^, CD11b^+^CD27^−^ (7). Compared to wild-type mice, *Kctd9*^−/−^ mice showed increased frequency of CD11b^−^CD27^+^ cells and reduced proportion of CD11b^+^CD27^−^ and/or CD11b^+^CD27^+^ subsets in CD3^−^DX5^+^ cells (Figures 3A,B), suggesting the impairment of NK cell maturation. This phenotype was further confirmed by the lower ratio of CD11b^+^ to CD11b^−^ NK cells in all organs in *Kctd9*^−/−^ mice (Figure 3C). Acquisition of CD11b represents a process of NK cell maturation that establishes primary effector function, whereas, loss of CD27 expression on CD11b^+^ NK cells predicts a terminal maturation with full effector function potential (5, 7). In *Kctd9*^−/−^ mice, the ratio of CD27^−^ subset to CD27^+^ subset among CD11b^+^ NK cells were remarkably decreased in the peripheral organs (Figure 3D). Using DX5 to label NK cells may result in the selection of non-NK cells such as basophils and mast cells (28). However, mast cells (FSC^high^ SSC^high^) may be subjected to gate restriction on lymphocytes (FSC^low^ SSC^low^) (28). Moreover, DX5^+^ CD3ε^−^ fraction includes about 90–95% NK cells and < 10% basophils in splenocytes (29). Thus, the use of either DX5 or NKp46 to determine splenic NK cell development and function is persuasive. To validate the results, NKp46^+^ NK cells were also analyzed to dissect bone marrow NK cell development. Results in terms of less mature NK cells (CD27^+^CD11b^−^) and mature NK cells (CD27^+^ CD11b^+^/CD27^−^CD11b^+^) were very similar to those defined by DX5, except that double negative NK cells were not altered (Figure 3E). Collectively, these data suggest that Kctd9 regulates the NK cells maturation.

### Kctd9 Restricts the Proliferation of NK Cells

**Figure 4:**
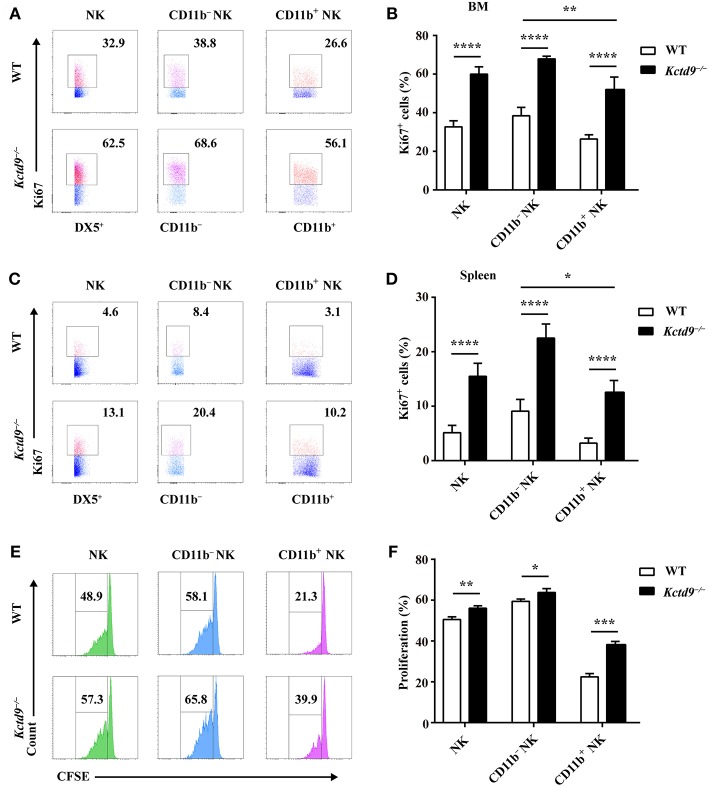
Kctd9 restricts the proliferation of NK cells. **(A–D)** Expression of Ki67 in NK cells and NK subsets in BM and spleen from WT and *Kctd9*^−/−^ mice. **(A**,**C)** Representative flow cytometric analysis for Ki67 expression in BM **(A)** and spleen **(C)**. Flow cytometric plots represent total CD3^−^DX5^+^ NK cells (left), CD11b^−^CD3^−^DX5^+^ NK subset (middle), and CD11b^+^CD3^−^DX5^+^ NK subset (right). Frequency of gated population is indicated. **(B**,**D)** Summary of the percentages of CD3^−^DX5^+^ NK cells, CD11b^−^CD3^−^DX5^+^ subset, and CD11b^+^CD3^−^DX5^+^ subset positive for Ki67 in indicated organs. **(E)** Flow cytometric analysis for CFSE concentration in purified splenic NK cells and CD11b^−^ NK subset and CD11b^+^ NK subset 72 h after initiation of culture in the presence of IL-15. **(F)** Summary of the percentages of NK cells and CD11b^−^ NK subset and CD11b^+^ NK subset with reduced concentration of CFSE. All results were representative of three independent experiments. Four mice of each genotype were used in each experiment. Error bars indicate standard deviation. *P < 0.05, **P < 0.01, ***P < 0.001, and ****P < 0.0001.

NK cell reduction may be due to altered proliferation or apoptosis. Therefore, to examine the proliferative potential of NK cells, NK cells from bone marrow and spleen were subjected to Ki67 staining. In *Kctd9*^−/−^ mice, the number of proliferating NK cells, including either CD11b^−^ or CD11b^+^ cells was significantly increased in the bone marrow (Figures 4A,B). Substantial increase in proliferating NK cells was also observed in *Kctd9*^−/−^ spleen (Figures 4C,D). Similar results were further observed in *Kctd9*^−/−^ NK cells in response to IL-15 stimulation (Figures 4E,F), which is required for NK cell proliferation and homeostasis via metabolic activation of mTOR *in vivo* (30). Together, these data suggest that Kctd9 negatively regulates NK cell proliferation.

### Kctd9 Deficiency Impairs NK Cell Effector Functions

**Figure 5:**
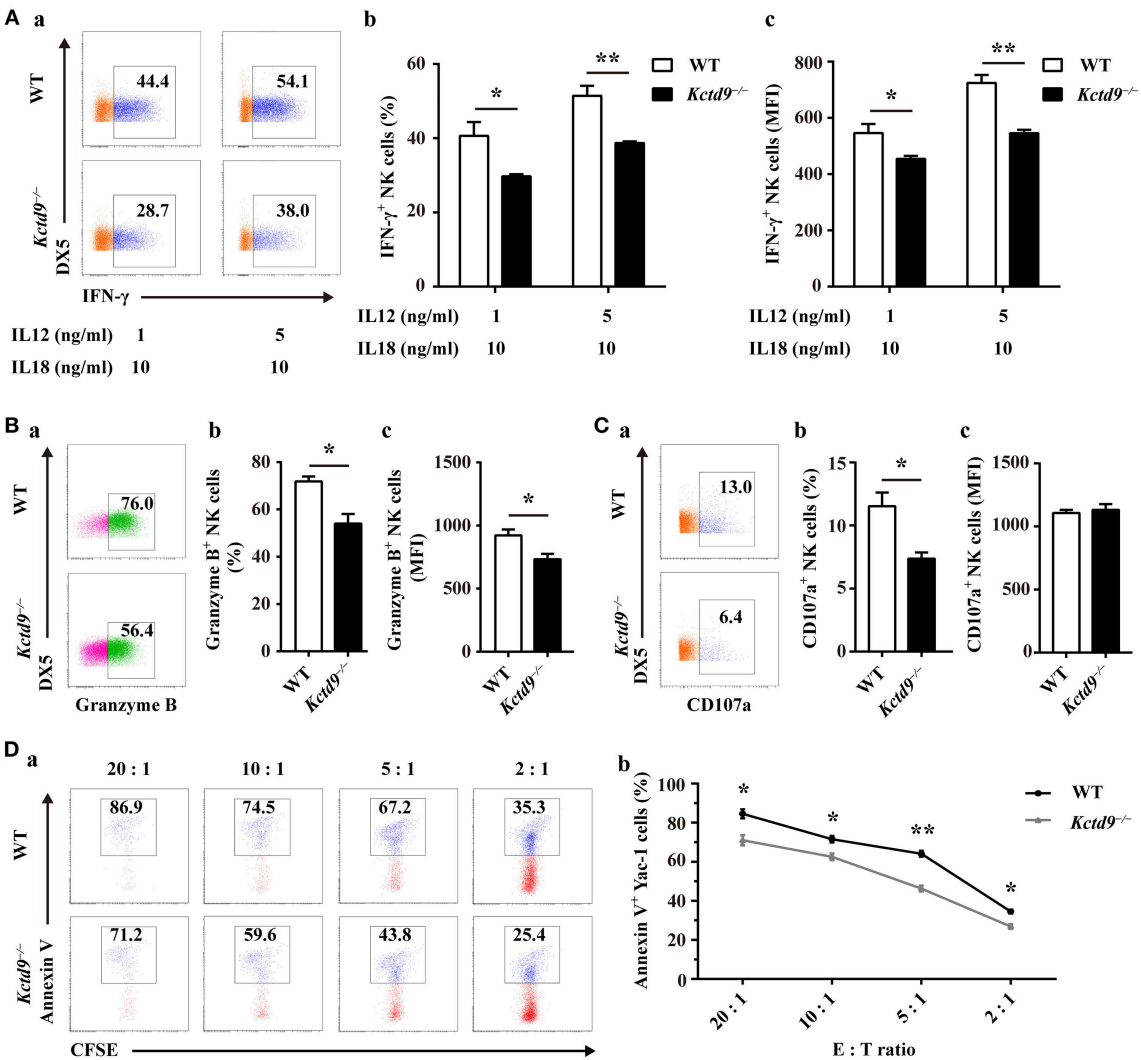
Kctd9 deficiency impairs NK cell effector functions. **(A–C)** Expression of IFN-γ, Granzyme B by NK cells and degranulation of NK cells from spleen of WT and *Kctd9*^−/−^ mice. *in vitro* cell activation is described in Materials and Methods. **(a)** Flow cytometric analysis for expression of IFN-γ, Granzyme B, and CD107a by CD3^−^DX5^+^ NK cells. Plots represent total CD3^−^DX5^+^ NK cells. Frequency of gated population is indicated in plots. **(b)** The percentage of CD3^−^DX5^+^ NK cells positive for IFN-γ **(A)**, Granzyme B **(B)**, and CD107a **(C)**. **(c)** The expression intensity of IFN-γ **(A)**, Granzyme B **(B)**, and CD107a **(C)** of gated CD3^−^DX5^+^ NK cells. **(D)** Apoptosis of Yac-1 cells after being co-cultured with purified NK cells from spleen of WT and *Kctd9*^−/−^ mice. **(a)** Flow cytometric analysis for Annexin V on Yac-1 cells. Plots represent CFSE^+^ Yac-1 cells. **(b)** Summary of the percentage of apoptotic Yac-1 cells positive for Annexin V. All results were representative of three independent experiments. Four to six mice of each genotype were used in each experiment. Error bars indicate standard deviation. *p < 0.05, **p < 0.01.

NK cell effector function is associated with the normal development and maturation of cells. To determine whether Kctd9 affects NK cell effector function, we examined IFN-γ production and cytotoxic potential of wild-type and *Kctd9*^−/−^ NK cells in response to cytokine stimulation or tumor cell challenge. After stimulation with IL-12 and IL-18, *Kctd9*^−/−^ NK cells displayed decreased portion of IFN-γ^+^ cells as well as lower level of IFN-γ (Figure 5A). The discrepancy between the expression of IFN-γ by *Kctd9*^−/−^ NK cells and wild-type NK cells was maintained in response to low IL-12 concentration (Figure 5A). Moreover, a reduced portion of Granzyme B-positive NK cells as well as lower intensity of Granzyme B expression were observed in *Kctd9*-deficient splenic NK cells in response to IL-15 treatment (Figure 5B). Consistently, a decreased proportion of *Kctd9*^−/−^ NK cells degranulated in response to IL-12/IL-18 treatment, though their CD107a level did not alter (Figure 5C). Furthermore, compared with wild-type NK cells, *Kctd9*^−/−^ splenic NK cells showed reduced cytotoxicity against Yac-1 *in vitro* (Figure 5D). These data suggest that Kctd9 deficiency attenuates NK cell effector functions.

### Kctd9 Depletion Downregulated NK Cell-Related Transcription Factor Expression

**Figure 6:**
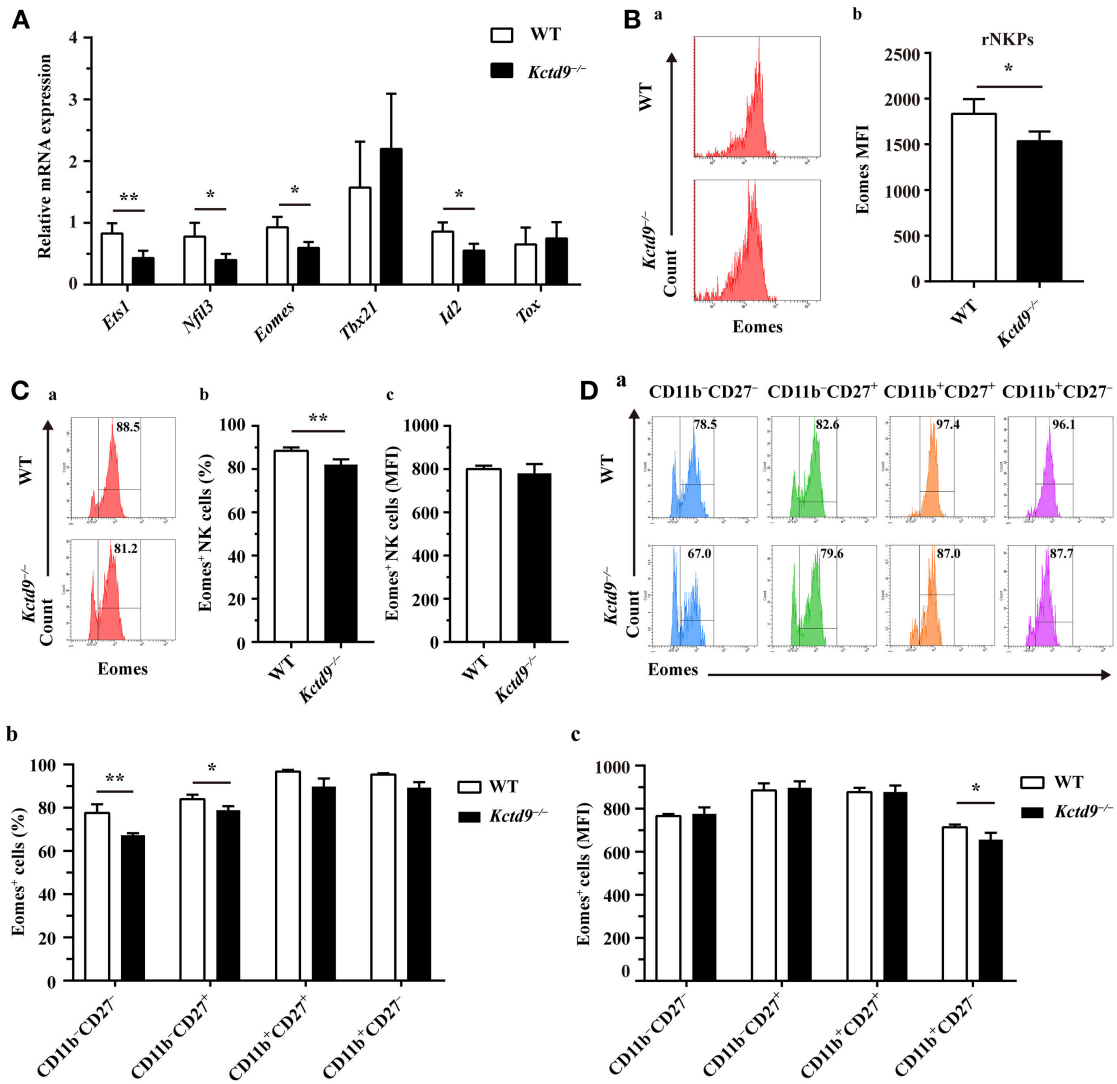
Kctd9 depletion downregulated NK cell-related transcription factor expression. **(A)** Real-time PCR analysis for *Ets1, Nfil3* (encodes E4bp4), *Eomes, Tbx21* (encodes T-bet), *Id2*, and *Tox* expression by purified NK cells from spleen of WT and *Kctd9*^−/−^ mice. **(B)** Expression of Eomes by rNKPs in BM from WT and *Kctd9*^−/−^ mice. **(a)** Flow cytometric analysis for Eomes in rNKPs. Plots represent rNKPs. **(b)** Intensity of Eomes expression in total rNKPs. **(C,D)** Expression of Eomes by CD3^−^NKp46^+^ NK cells **(C)** and subsets defined by CD11b/CD27 expression in CD3^−^NKp46^+^ NK cells **(D)** from WT and *Kctd9*^−/−^ BM. **(a)** Flow cytometric analysis for expression of Eomes. Plots represent CD3^−^NKp46^+^ NK cells **(C)** or subsets of CD3^−^NKp46^+^ NK cells **(D)**. Frequency of gated population is indicated in plots. **(b,c)** The percentages and the intensity of Eomes expression of CD3^−^NKp46^+^ NK cells **(C)** and subsets of CD3^−^NKp46^+^ NK cells **(D)** positive for Eomes. All results were representative of three independent experiments. Four to six mice of each genotype were used in each experiment. Error bars indicate standard deviation. *P < 0.05, **p < 0.01.

NK cell development is tightly regulated by extrinsic signals and intrinsic transcription factors. Factors involved in NK cell early development, such as Nfil3 and Ets1, and maturation, such as Eomes and Id2, were expressed in lower level by *Kctd9*^−/−^ NK cells than wild-type NK cells (Figure 6A). Owing to the selective reduction of rNKPs as well as NK cells in bone marrow of *Kctd9*^−/−^ mice, which rely on CD122 expression, we investigated whether Kctd9 regulates CD122 expression in NK cells. However, we did not observe a decline in CD122 expression in rNKPs (data not shown), implying that Kctd9 may be involved in regulating intrinsic transcriptional factors.

Eomes and T-bet, members of the T-box family of proteins, were demonstrated to control NK cell development from immature to mature phenotype, and mature stage to terminal maturation, respectively (16). Eomes induces the expression of NK-antigen receptors during NK cell maturation, whereas T-bet controls the repression of CD27 and induction of KLRG1 and CD43 expression for terminal maturation of CD11b^+^ NK cells (16). In addition to a reduction of Eomes expression in rNKPs (Figure 6B), a decreased proportion of NK cells from *Kctd9*^−/−^ bone marrow expressed Eomes though the positive population maintained unaltered mean expression intensity (Figure 6C). The decrease of Eomes^+^NKp46^+^ NK cells was mostly in CD11b^−^ NK cells (Figure 6D), and Eomes MFI was decreased in CD11b^+^CD27^−^ NK cells (Figure 6D). These data suggest that Kctd9 depletion downregulated the expression of the transcription factors required for NK cell commitment and maturation.

## Discussion

NK cell development and maturation are associated with the sequential acquisition and loss of surface antigens, as well as spatiotemporal transactivation of transcriptional factors. Kctd9 deficiency selectively reduces the number of rNKPs, a phenotype resembling the observation in *Ets*^−/−^ mice, implying that Kctd9 is involved in NK cell lineage commitment and maturation by regulating such factors. Transcription factors such as E4BP4/NFIL3 and ETS1 define NK cell lineage commitment with an unknown mechanism; however, their involvement in NK cell maturation is mediated by regulating Eomes and ID2 or T-bet/Tbx21 and ID2, respectively (14, 15). Expression of Eomes leads to demarcation of two subsets of NK cells: less mature (Eomes^−^ TRAIL^+)^ NK cells and more mature (Eomes^+^DX5^+^) NK cells with a diverse repertoire of Ly49 receptors (16), whereas Tbx21-deficient mice lack Eomes^−^TRAIL^+^ NK cells. We found that Kctd9 affect expression of E4BP4/NFIL3 and ETS1 as well as Eomes and ID2 by NK cells. Further studies are warranted to identify the precise molecular targets of Kctd9 in NK cells.

The use of DX5 to define NK cells may not be stringent, particularly for immature CD11b^−^CD27^−^ NK cells in the bone marrow, because such cells account for a small fraction (<10%) and may include basophils (CD11b^dull^ CD27^−^) (7, 31, 32). However, basophils only account for small fraction (~7%) of CD3ε^−^DX5^+^ cells in the spleen and circulating nucleated cells (29), paving the way to use DX5 to identify splenic NK cells for development, proliferation assay, and cytotoxicity assays. Moreover, NKp46^+^ NK cells account for nearly 90% of CD3ε^−^ DX5^+^ cells in the spleen, confirming that the majority of CD3ε^−^ DX5^+^ are NK cells (Supplementary Figure 1D). Because CD3ε^−^ DX5^+^ cells may include a relatively large fraction of non-NK cells in the bone marrow, NKp46 was also employed to dissect NK cell development. As depicted in Figure 3E, NK cell development was inhibited. NK cell development driven by Kctd9 may be intrinsic and/or extrinsic. As CD4^+^ and CD8^+^ T cells were also decreased in *Kctd9*^−/−^ bone marrow, alteration in extrinsic factors for NK cell development in the bone marrow cannot be excluded (Supplementary Figures 1F–H).

Moreover, in contrast to the positive role of *Kctd9* in NK cell development and effector functions, Kctd9 inhibits proliferation of NK cells in the steady state and under cytokine stimulation. These opposite roles of Kctd9 in NK cell development and proliferation are consistent with the fact that the proliferative capacity of NK cells gradually declines as they mature (7, 9, 33). The increased proliferative capacity of *Kctd9*^−/−^ NK cells may be due to their immature status rather than direct inhibition of proliferation by Kctd9. The immature status of *Kctd9*^−/−^ NK cells may also account for the increase of NK cells in the liver (Figure 2E) and impaired effector functions in response to stimulation by cytokines, exogenous antigens, or even viral infection.

KCTD9 contains the BTB/POZ domain which could interact with CUL3 (an E3 ligase) for target protein ubiquitination and degradation (34, 35). The CUL3-RING ubiquitin ligase complex associates with BTB adaptors, which enable homodimerization, assembly, and substrate recognition, thereby specifying the substrate selectivity of E3 ligase (36, 37). BTB proteins include KLHL proteins and SPOP (MATH-BTB-BACK domain) proteins (37), PLZF (BTB-Zinc finger) (38), and KCTD proteins (voltage-gated potassium channel T1 family proteins) (39). Physical interaction between KCTD9 and CUL3 has been experimentally confirmed via cryo-electron microscopy (39), implying that the ubiquitination-based mechanism of CUL3 is likely dependent on KCTD9 partners. In addition to the truncated T1 domain that contains BTB, a unique eukaryotic-specific DUF3354 domain and eight partial overlapping pentapeptide repeats are present in KCTD9 (21). The function of DUF3354 is unknown. The pentapeptide repeats, which mimic DNA structure, can interact with DNA-binding proteins, such as DNA gyrase or DNA polymerase (34). Therefore, the effects of Kctd9 in NK cell development and function may be mediated by direct or indirect interaction-dependent degradation of transcription factors or chromatin regulators critical for NK cell commitment and maturation. This process resembles the involvement of PLZF in lymphoid effector programs wherein PLZF bridges between CUL3 and substrates such as chromatin modifiers, nuclear transporters, and members of the transcriptional machinery (38). Therefore, we speculate that understanding the binding partners of Kctd9 in NK cells will pave the way to understanding the mechanism underlying Kctd9-mediated regulation of the NK cell program and host defense against pathogens and malignancies.

Viral fulminant hepatitis is a type of acute liver failure with high mortality. Hepatic recruitment and activation of NK cells accelerate inflammation accumulation and confer cell contact-dependent or independent cytotoxicity in both patients and experimental models of hepatitis virus-induced acute liver damage (25, 40–42). Our findings provide a new insight on the choice of NK-cell based therapeutic targets in viral fulminant hepatitis.

In summary, we propose that Kctd9 is required for NK cell development and effector function, and it plays a detrimental role in inflammation-induced fulminant hepatitis. The mechanism may involve CUL3-based ubiquitination and degradation of Kctd9 partners, which are essential for the early and/or late stages of NK cell development and effector function. This study highlights the novel therapeutic and preventive implications for acute severe liver damage in hepatitis.

## Ethics Statement

This study was carried out in accordance with the recommendations of the guidelines of the National Institutes of Health and the Animal Experiment Committee of Tongji hospital. This study was reviewed and approved by the Animal Experiment Committee of Tongji hospital.

## Author Contributions

XW and XZ drafted the manuscript and interpreted data. XZ did the experiments. PW raised the animals. XG assisted collecting samples. QN, XW, TC, WY, PW, and XL assisted interpretation of data. QN and XW developed concept, designed projects. QN, XW, and GS revised manuscript.

### Conflict of Interest Statement

The authors declare that the research was conducted in the absence of any commercial or financial relationships that could be construed as a potential conflict of interest.

## Supplementary Material

Data Sheet 1 The detailed description of methodologies and the list of antibodies and primers used in this study.

Supplementary Figure 1 **(A)** Information of Kctd9-deficient mice. Sequencing results of PCR products amplified from total DNA template of WT mice and *Kctd9*^−/−^ mice were aligned using Seqman software. Sequencing alignment between truncated *Kctd9* sequence in *Kctd9*^−/−^ mice and wild-type (WT) *Kctd9* sequence (left top). Schematic view of deleted sequence which includes 40 nucleotides in the open reading frame in exon 6 and 30 nucleotides in intron 6 of *Kctd9* allele (left bottom). Immunoblotting against Kctd9 protein of splenocytes from WT and *Kctd9*^−/−^ mice (Right). **(B)** Total BM cell number of WT mice and *Kctd9*^−/−^ mice. **(C)** Proportion of BM rNKPs among Lin^−^CD244^+^CD27^+^CD127^+^ population in WT mice and *Kctd9*^−/−^. **(D)** Proportions of NKp46^+^ cells among CD3^−^DX5^+^ cells in four organs from WT mice and *Kctd9*^−/−^ mice. **(E)** Total number of CD3^−^CD122^+^NKp46^+^DX5^+^cells in different organs from WT mice and *Kctd9*^−/−^ mice. **(F–H)** Total number of CD3^−^CD4^−^CD8^−^B220^+^ B cells, CD3^+^CD4^+^ T, and CD3^+^CD8^+^ T cells in different organs of WT mice and *Kctd9*^−/−^ mice. 3 *Kctd9*^−/−^ mice and 2 WT mice were used for Western Blotting. Six to eight mice of each genotype were used in other experiments. All results were representative of three independent experiments. Error bars indicate standard deviation. ^*^*P* < 0.05, ^**^*p* < 0.01, ^***^*P* < 0.001, and ^****^*P* < 0.0001.

## Abbreviations

KCTD9: potassium channel tetramerization domain containing 9
NK cells: natural killer cell
HBV-ACLF: hepatitis B virus-associated acute-on-chronic liver failure
MHV-3-FHF: murine hepatitis virus strain 3-induced fulminant hepatic failure
MHV-3: murine hepatitis virus strain 3
WT: wild-type
BM: bone marrow
CLPs: common lymphoid progenitors
NKPs: NK progenitors
rNKPs: refined NKPs
iNK cells: immature NK cells
mNK cells: mature NK cells.

## References

[1] Geiger TL Sun JC. Development and maturation of natural killer cells. Curr Opin Immunol. (2016) 39:82–9. 10.1016/j.coi.2016.01.007 26845614 PMC4801705

[2] Rosmaraki EE Douagi I Roth C Colucci F Cumano A Di Santo JP. Identification of committed NK cell progenitors in adult murine bone marrow. Eur J Immunol. (2001) 31:1900–9. 10.1002/1521-4141(200106)31:6<60;1900::AID-IMMU1900>62;3.0.CO;2-M 11433387

[3] Carotta S Pang SH Nutt SL Belz GT. Identification of the earliest NK-cell precursor in the mouse BM. Blood. (2011) 117:5449–52. 10.1182/blood-2010-11-318956 21422472

[4] Fathman JW Bhattacharya D Inlay MA Seita J Karsunky H Weissman IL. Identification of the earliest natural killer cell-committed progenitor in murine bone marrow. Blood. (2011) 118:5439–47. 10.1182/blood-2011-04-348912 21931117 PMC3217348

[5] Kim S Iizuka K Kang HS Dokun A French AR Greco S. *In vivo* developmental stages in murine natural killer cell maturation. Nat Immunol. (2002) 3:523–8. 10.1038/ni796 12006976

[6] Vosshenrich CA Ranson T Samson SI Corcuff E Colucci F Rosmaraki EE. Roles for common cytokine receptor gamma-chain-dependent cytokines in the generation, differentiation, and maturation of NK cell precursors and peripheral NK cells *in vivo*. J Immunol. (2005) 174:1213–21. 10.4049/jimmunol.174.3.1213 15661875

[7] Chiossone L Chaix J Fuseri N Roth C Vivier E Walzer T. Maturation of mouse NK cells is a 4-stage developmental program. Blood. (2009) 113:5488–96. 10.1182/blood-2008-10-187179 19234143

[8] Walzer T Chiossone L Chaix J Calver A Carozzo C Garrigue-Antar L. Natural killer cell trafficking *in vivo* requires a dedicated sphingosine 1-phosphate receptor. Nat Immunol. (2007) 8:1337–44. 10.1038/ni1523 17965716

[9] Hayakawa Y Smyth MJ. CD27 dissects mature NK cells into two subsets with distinct responsiveness and migratory capacity. J Immunol. (2006) 176:1517–24. 10.4049/jimmunol.176.3.1517 16424180

[10] Huntington ND Tabarias H Fairfax K Brady J Hayakawa Y Degli-Esposti MA. NK cell maturation and peripheral homeostasis is associated with KLRG1 up-regulation. J Immunol. (2007) 178:4764–70. 10.4049/jimmunol.178.8.4764 17404256

[11] DiSanto JP Muller W Guy-Grand D Fischer A Rajewsky K. Lymphoid development in mice with a targeted deletion of the interleukin 2 receptor gamma chain. Proc Natl Acad Sci USA. (1995) 92:377–81. 7831294 10.1073/pnas.92.2.377 PMC42743

[12] Kennedy MK Glaccum M Brown SN Butz EA Viney JL Embers M. Reversible defects in natural killer and memory CD8 T cell lineages in interleukin 15-deficient mice. J Exp Med. (2000) 191:771–80. 10.1084/jem.191.5.771 10704459 PMC2195858

[13] Luevano M Madrigal A Saudemont A. Transcription factors involved in the regulation of natural killer cell development and function: an update. Front Immunol. (2012) 3:319. 10.3389/fimmu.2012.00319 23087693 PMC3470934

[14] Male V Nisoli I Kostrzewski T Allan DS Carlyle JR Lord GM. The transcription factor E4bp4/Nfil3 controls commitment to the NK lineage and directly regulates Eomes and Id2 expression. J Exp Med. (2014) 211:635–42. 10.1084/jem.20132398 24663216 PMC3978281

[15] Ramirez K Chandler KJ Spaulding C Zandi S Sigvardsson M Graves BJ. Gene deregulation and chronic activation in natural killer cells deficient in the transcription factor ETS1. Immunity. (2012) 36:921–32. 10.1016/j.immuni.2012.04.006 22608498 PMC3389314

[16] Gordon SM Chaix J Rupp LJ Wu J Madera S Sun JC. The transcription factors T-bet and Eomes control key checkpoints of natural killer cell maturation. Immunity. (2012) 36:55–67. 10.1016/j.immuni.2011.11.016 22261438 PMC3381976

[17] Boos MD Yokota Y Eberl G Kee BL. Mature natural killer cell and lymphoid tissue-inducing cell development requires Id2-mediated suppression of E protein activity. J Exp Med. (2007) 204:1119–30. 10.1084/jem.20061959 17452521 PMC2118569

[18] Aliahmad P de la Torre B Kaye J. Shared dependence on the DNA-binding factor TOX for the development of lymphoid tissue-inducer cell and NK cell lineages. Nat Immunol. (2010) 11:945–52. 10.1038/ni.1930 20818394 PMC2943551

[19] Vosshenrich CA Garcia-Ojeda ME Samson-Villeger SI Pasqualetto V Enault L Richard-Le Goff O. A thymic pathway of mouse natural killer cell development characterized by expression of GATA-3 and CD127. Nat Immunol. (2006) 7:1217–24. 10.1038/ni1395 17013389

[20] Sojka DK Plougastel-Douglas B Yang L Pak-Wittel MA Artyomov MN Ivanova Y. Tissue-resident natural killer (NK) cells are cell lineages distinct from thymic and conventional splenic NK cells. Elife. (2014) 3:e01659. 10.7554/eLife.01659 24714492 PMC3975579

[21] Skoblov M Marakhonov A Marakasova E Guskova A Chandhoke V Birerdinc A. Protein partners of KCTD proteins provide insights about their functional roles in cell differentiation and vertebrate development. Bioessays. (2013) 35:586–96. 10.1002/bies.201300002 23592240

[22] Zhou YY Zou Y Chen T Wang HW Han MF Pi B. [KCTD9, a novel potassium channel related gene, was highly expressed in hepatic NK cells and T cells of fulminant hepatitis mice induced by MHV-3]. Zhonghua Gan Zang Bing Za Zhi. (2011) 19:833–7. 10.3760/cma.j.issn.1007-3418.2011.11.010 22433305

[23] Chen T Zhu L Zhou Y Pi B Liu X Deng G. KCTD9 contributes to liver injury through NK cell activation during hepatitis B virus-induced acute-on-chronic liver failure. Clin Immunol. (2013) 146:207–16. 10.1016/j.clim.2012.12.013 23376586

[24] Yang M Chen S Du J He J Wang Y Li Z. NK cell development requires Tsc1-dependent negative regulation of IL-15-triggered mTORC1 activation. Nat Commun. (2016) 7:12730. 10.1038/ncomms12730 27601261 PMC5023956

[25] Zou Y Chen T Han M Wang H Yan W Song G. Increased killing of liver NK cells by Fas/Fas ligand and NKG2D/NKG2D ligand contributes to hepatocyte necrosis in virus-induced liver failure. J Immunol. (2010) 184:466–75. 10.4049/jimmunol.0900687 19949088

[26] Marsden PA Ning Q Fung LS Luo X Chen Y Mendicino M. The Fgl2/fibroleukin prothrombinase contributes to immunologically mediated thrombosis in experimental and human viral hepatitis. J Clin Investig. (2003) 112:58–66. 10.1172/JCI18114 12840059 PMC162293

[27] Walzer T Blery M Chaix J Fuseri N Chasson L Robbins SH. Identification, activation, and selective *in vivo* ablation of mouse NK cells via NKp46. Proc Natl Acad Sci USA. (2007) 104:3384–9. 10.1073/pnas.0609692104 17360655 PMC1805551

[28] Gessner A Mohrs K Mohrs M. Mast cells, basophils, and eosinophils acquire constitutive IL-4 and IL-13 transcripts during lineage differentiation that are sufficient for rapid cytokine production. J Immunol. (2005) 174:1063–72. 10.4049/jimmunol.174.2.1063 15634931

[29] Nishikado H Mukai K Kawano Y Minegishi Y Karasuyama H. NK cell-depleting anti-asialo GM1 antibody exhibits a lethal off-target effect on basophils *in vivo*. J Immunol. (2011) 186:5766–71. 10.4049/jimmunol.1100370 21490162

[30] Marcais A Cherfils-Vicini J Viant C Degouve S Viel S Fenis A. The metabolic checkpoint kinase mTOR is essential for IL-15 signaling during the development and activation of NK cells. Nat Immunol. (2014) 15:749–57. 10.1038/ni.2936 24973821 PMC4110708

[31] Dijkstra D Meyer-Bahlburg A. Human Basophils Modulate Plasma Cell Differentiation and Maturation. J Immunol. (2017) 198:229–38. 10.4049/jimmunol.1601144 27852746

[32] Mukai K Matsuoka K Taya C Suzuki H Yokozeki H Nishioka K. Basophils play a critical role in the development of IgE-mediated chronic allergic inflammation independently of T cells and mast cells. Immunity. (2005) 23:191–202. 10.1016/j.immuni.2005.06.011 16111637

[33] Goh W Huntington ND. Regulation of Murine Natural Killer Cell Development. Front Immunol. (2017) 8:130. 10.3389/fimmu.2017.00130 28261203 PMC5309223

[34] Merens A Matrat S Aubry A Lascols C Jarlier V Soussy CJ. The pentapeptide repeat proteins MfpAMt and QnrB4 exhibit opposite effects on DNA gyrase catalytic reactions and on the ternary gyrase-DNA-quinolone complex. J Bacteriol. (2009) 191:1587–94. 10.1128/JB.01205-08 19060136 PMC2648189

[35] Xu L Wei Y Reboul J Vaglio P Shin TH Vidal M. BTB proteins are substrate-specific adaptors in an SCF-like modular ubiquitin ligase containing CUL-3. Nature. (2003) 425:316–21. 10.1038/nature01985 13679922

[36] Geyer R Wee S Anderson S Yates J Wolf DA. BTB/POZ domain proteins are putative substrate adaptors for cullin 3 ubiquitin ligases. Mol Cell. (2003) 12:783–90. 10.1016/S1097-2765(03)00341-1 14527422

[37] Canning P Cooper CD Krojer T Murray JW Pike AC Chaikuad A. Structural basis for Cul3 protein assembly with the BTB-Kelch family of E3 ubiquitin ligases. J Biol Chem. (2013) 288:7803–14. 10.1074/jbc.M112.437996 23349464 PMC3597819

[38] Mathew R Seiler MP Scanlon ST Mao AP Constantinides MG Bertozzi-Villa C. BTB-ZF factors recruit the E3 ligase cullin 3 to regulate lymphoid effector programs. Nature. (2012) 491:618–21. 10.1038/nature11548 23086144 PMC3504649

[39] Ji AX Chu A Nielsen TK Benlekbir S Rubinstein JL Prive GG. Structural insights into KCTD protein assembly and Cullin3 recognition. J Mol Biol. (2016) 428:92–107. 10.1016/j.jmb.2015.08.019 26334369

[40] Amadei B Urbani S Cazaly A Fisicaro P Zerbini A Ahmed P. Activation of natural killer cells during acute infection with hepatitis C virus. Gastroenterology. (2010) 138:1536–45. 10.1053/j.gastro.2010.01.006 20080094 PMC4183834

[41] Zhao J Li Y Jin L Zhang S Fan R Sun Y. Natural killer cells are characterized by the concomitantly increased interferon-gamma and cytotoxicity in acute resolved hepatitis B patients. PLoS ONE. (2012) 7:e49135. 10.1371/journal.pone.0049135 23133672 PMC3486810

[42] Okazaki A Hiraga N Imamura M Hayes CN Tsuge M Takahashi S. Severe necroinflammatory reaction caused by natural killer cell-mediated Fas/Fas ligand interaction and dendritic cells in human hepatocyte chimeric mouse. Hepatology. (2012) 56:555–66. 10.1002/hep.25651 22331638

