# Supplementary material for: Kctd9 Deficiency Impairs Natural Killer Cell Development and Effector Function
Source: Front Immunol. 2019 Apr 10;10:744. doi: 10.3389/fimmu.2019.00744 (PMC6467973; doi:10.3389/fimmu.2019.00744)
Supplement: Data Sheet 1 — The detailed description of methodologies and the list of antibodies and primers used in this study. [file Data_Sheet_1.docx]

**Isolation of lymphocytes in the liver**

Liver tissue were cut into pieces, and grinded on pre-moisturized 100 μm cell strainer (BD Biosciences) accompanied by washing with RPMI 1640 medium. Cells from liver were resuspended in 40% Percoll plus (cat# E0414, Sigma) solution. Layered cell suspension onto 70% Percoll plus solution, and centrifuged at 800×g for 20 min. Collected lymphocytes from the interphase. For cell number assay, cell suspension was directly centrifuged in 40% Percoll plus at 800×g for 20 min and pellet in the bottom was left. The pellet was resuspended in ACK lysis buffer to remove red cells.

**Antibodies**

Antibodies against mouse CD3e FITC (clone 145-2C11, cat# 553062) or PE-Cy7 (cat# 561100), CD19 FITC (clone 1D3, cat# 553785), CD11b FITC (clone M1/70, cat# 557396) or PE (cat# 557397), CD49b FITC (clone: DX5, cat# 553857) or APC (cat# 560628), CD27 PerCP-Cy5.5 (clone LG.3A10, cat# 563603), CD127 PE-Cy7 (clone SB/199, cat# 560733), CD122 PerCP-Cy5.5 (clone TM-β1, cat# 564764), TER-119 FITC (clone TER-119, cat# 557915), CD335 PerCP-Cy5.5 (clone 29A1.4, cat# 560800), Ly49C/I PE (clone 5E6, cat# 553277), Ly-49G2 PE-Cy7 (clone 4D11, cat# 560730), Ki-67 PE-Cy7 (clone B56, cat# 561283), IFN-γ PE (clone XMG1.2, cat# 554412) and isotype control antibodies were purchased from BD Biosciences. Antibodies against mouse CD3e FITC (clone 145-2C11, cat# 100306) or PE/Cy7 (cat# 100320), CD49b FITC (clone DX5, cat# 108906), Ly-6D FITC (clone 49-H4, cat# 138606), CD27 APC/Cy7 (clone LG.3A10, cat# 124225), CD135 PE (clone A2F10, cat# 135306),CD122 PerCP/Cy5.5 (clone TM-β1, cat# 123212), CD4 FITC (clone GK1.5, cat# 100406), CD8a FITC (clone 53-6.7, cat#: 100706), TER-119 FITC (clone TER-119, cat# 116206), KLRG1 PE/Cy7 (clone 2F1/KLRG1, cat# 138416), CD107 PerCP/Cy5.5 (clone 1D4B, cat# 121625), Granzyme B FITC (clone GB11, cat# 515403) and isotype control antibodies were purchased from BioLegend. Antibodies against mouse CD244.1 APC (clone C9.1, cat# 17-2440-82), CD11b PerCP-Cy5.5 (clone M1/70, cat# 45-0112-82), EOMES PE (clone Dan11mag, cat# 12-4875-80) and isotype control antibodies were purchased from ThermoFisher Scientific. Antibody against mouse NKG2A/CD159a PE (clone 705829, cat# FAB6867P-025) and isotype control antibody were purchased from R&D.

**Antibodies staining**

Cells were incubated with antibody cocktail against surface markers in Stain Buffer (FBS) (cat# 554656, BD Biosciences). Cells were fixed and permeabilized, and then subjected to intracellular staining with antibodies against intracellular molecules. Cytofix/Cytoperm Fixation/Permeabilization solution (cat# 554714, BD Bioscience) was used for permeabilization and staining with antibodies against IFN-γ or Granzyme B. Transcription Factor Staining Buffer (cat#: 00-5523-00, ThermoFisher Scientific) was applied to permeabilization and staining with antibodies against Ki67 and Eomes.

**Western Blotting**

Splenocytes from both WT and *Kctd9*^−^*^/^*^−^ mice were lysed in RIPA buffer. Cell lysates were boiled and loaded to gel for SDS-PAGE. Primary antibody against KCTD9(cat# sc-87182, Santa Cruz) was incubated with protein-loaded PVDF membrane followed by washing with TBST for three times. Antibody-stained membrane was incubated with HRP-conjugated Rabbit anti-goat IgG (cat#：BA1060, Boster) and subjected to ECL Western Blotting Substrate reaction. The membrane was subjected to Chemiluminescence. The KCTD9 antibody (Cat# sc-87182, Santa Cruz,), is an affinity purified goat polyclonal antibody raised against a peptide mapping at the C-terminus of KCTD9 of human origin. As instructed, this antibody can react both human and mouse Kctd9 protein in a western blot analysis.

**Primers**

We used the following primers:

| Gene | Forward primer | Reverse primer | Application |
| --- | --- | --- | --- |
| *Kctd9* | AGATCATAGAGGAGCATT | CTGTAAGCCCAAATCAA | Genotyping |
| *Ets1* | TCCTATCAGCTCGGAAGAACTC | TCTTGCTTGATGGCAAAGTAGTC | RT-PCR |
| *Nfil3* | CTGCGATGGTAGCCGGAAG | CGAGGACACCTCTGACACAT |  |
| *Eomes* | GCGCATGTTTCCTTTCTTGAG | GGTCGGCCAGAACCACTTC |  |
| *Tbx21* | AGCAAGGACGGCGAATGTT | GGGTGGACATATAAGCGGTTC |  |
| *Id2* | ATGAAAGCCTTCAGTCCGGTG | AGCAGACTCATCGGGTCGT |  |
| *Tox* | GCTCCCGTTCCATCCACAAA | TCCCAATCTCTTGCATCACAGA |  |
| *Gapdh* | AGGTCGGTGTGAACGGATTTG | TGTAGACCATGTAGTTGAGGTCA |  |
