# Supplementary figures and images for: Kctd9 Deficiency Impairs Natural Killer Cell Development and Effector Function
Source: Front Immunol. 2019 Apr 10;10:744. doi: 10.3389/fimmu.2019.00744 (PMC6467973; doi:10.3389/fimmu.2019.00744)

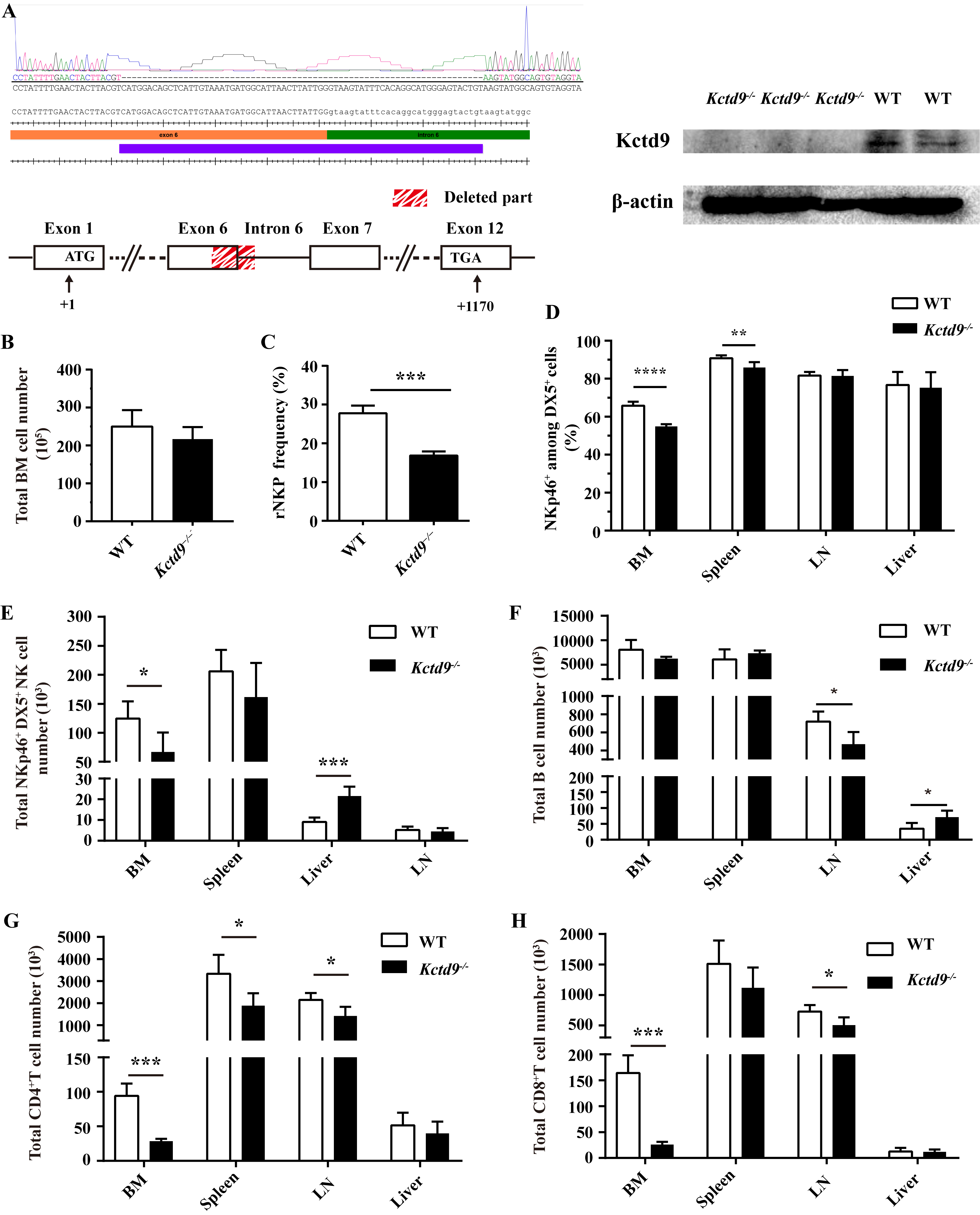

Supplement: Supplementary Figure 1 — (A) Information of Kctd9-deficient mice. Sequencing results of PCR products amplified from total DNA template of WT mice and Kctd9−/− mice were aligned using Seqman software. Sequencing alignment between truncated Kctd9 sequence in Kctd9−/− mice and wild-type (WT) Kctd9 sequence (left top). Schematic view of deleted sequence which includes 40 nucleotides in the open reading frame in exon 6 and 30 nucleotides in intron 6 of Kctd9 allele (left bottom). Immunoblotting against Kctd9 protein of splenocytes from WT and Kctd9−/− mice (Right). (B) Total BM cell number of WT mice and Kctd9−/− mice. (C) Proportion of BM rNKPs among Lin−CD244+CD27+CD127+ population in WT mice and Kctd9−/−. (D) Proportions of NKp46+ cells among CD3−DX5+ cells in four organs from WT mice and Kctd9−/− mice. (E) Total number of CD3−CD122+NKp46+DX5+cells in different organs from WT mice and Kctd9−/− mice. (F–H) Total number of CD3−CD4−CD8−B220+ B cells, CD3+CD4+ T, and CD3+CD8+ T cells in different organs of WT mice and Kctd9−/− mice. 3 Kctd9−/− mice and 2 WT mice were used for Western Blotting. Six to eight mice of each genotype were used in other experiments. All results were representative of three independent experiments. Error bars indicate standard deviation. *P < 0.05, **p < 0.01, ***P < 0.001, and ****P < 0.0001. [file Image_1.TIF]
